# Carotid artery elasticity decreases during pregnancy - the Cardiovascular Risk in Young Finns study

**DOI:** 10.1186/1471-2393-14-98

**Published:** 2014-03-06

**Authors:** Henna Kärkkäinen, Heli Saarelainen, Pirjo Valtonen, Tiina Laitinen, Olli T Raitakari, Markus Juonala, Mika Kähönen, Nina Hutri-Kähönen, Seppo Heinonen, Tomi Laitinen

**Affiliations:** 1 Department of Obst/Gyn, Kuopio University Hospital, University of Eastern Finland, P.O.B. 100FIN-70029 Kuopio, Finland; 2 Eastern Finland Laboratory Centre Joint Authority Enterprise (ISLAB), Mikkeli Regional Laboratory, Mikkeli, Finland; 3 Department of Clinical Physiology and Nuclear Medicine, Kuopio University Hospital, University of Eastern Finland, Kuopio, Finland; 4 Research Centre of Applied and Preventive Cardiovascular Medicine, University of Turku, Turku, Finland; 5 Department of Clinical Physiology and Nuclear Medicine, Turku University Hospital, Turku, Finland; 6 Department of Internal Medicine, Turku University Hospital, Turku, Finland; 7 Department of Clinical Physiology, University of Tampere and Tampere University Hospital, Tampere, Finland; 8 Department of Pediatrics, University of Tampere and Tampere University Hospital, Tampere, Finland

**Keywords:** Carotid artery, Elasticity, Pregnancy, Distensibility, Arterial stiffness, The Cardiovascular Risk in Young Finns study

## Abstract

**Background:**

The aims were to evaluate the effect of pregnancy on carotid artery elasticity and determine the associations between maternal lipids, endothelial function and arterial elasticity during pregnancy.

**Methods:**

We examined 99 pregnant and 99 matched non-pregnant control women as part of a population-based prospective cohort study. Carotid artery elasticity indexes; carotid artery distensibility (CAD), Young’s elastic modulus (YEM) and stiffness index (SI) as well as brachial artery flow-mediated dilation (FMD) were assessed using ultrasound; serum lipid levels were also determined.

**Results:**

SI was 57% and YEM 75% higher and CAD 36% lower in the third trimester group than the corresponding values in the first trimester group. Serum cholesterol and triglyceride levels were significantly higher in women at the end of the pregnancy than at the beginning of pregnancy (P < 0.001) and in controls (P < 0.001). In multivariate analysis, gestational age was the only independent correlate of arterial elasticity in pregnant women. In controls, age (P ≤ 0.001) and common carotid diameter (P = 0.001-0.029) were associated with SI, YEM and CAD.

**Conclusions:**

The present study revealed that carotid artery elasticity declined towards the end of the pregnancy; this neither is straight correlating with maternal hyperlipidemia or the diameter of the carotid artery nor is it associated with changes in endothelial function.

## Background

During pregnancy, characteristic changes occur in hemodynamic function: total peripheral resistance falls whereas plasma volume, stroke volume, cardiac output and heart rate increases. In non-pregnant subjects, arterial stiffness is a strong predictor of the risk of suffering cardiovascular events. Arterial stiffness during uncomplicated pregnancy has been examined with many techniques with various results [1-4] and particularly during hypertonic pregnancies [5,6]. The current concept is that during an uncomplicated pregnancy, arterial distensibility increases whereas in preeclampsia, arterial distensibility is reduced and arterial stiffness is elevated. However, changes in arterial stiffness are not similar in the whole arterial tree, but are regionally differentiated. Changes in the elastic properties of the carotid artery are thought to be different from those occurring in the aorta during pregnancy, and the carotid artery stiffens independently of other arterial beds [7]. In fact, a Hungarian study group has claimed that all carotid artery elastic parameters were adversely affected during pregnancy [8].

We and others have shown that there is a significant elevation in all lipid levels during pregnancy [9-11]. The changes in maternal cholesterol and triglyceride levels are thought to be physiological due to the needs of embryonic and fetal development but exaggerated lipid levels have also been linked to pathological pregnancies such as gestational diabetes mellitus [12]. We have also demonstrated that pregnancy contributes to brachial artery flow-mediated dilation (FMD) suggesting that vascular endothelial function is altered during pregnancy [11]. We hypothesized that both pregnancy-related changes, maternal hyperlipidemia and enhanced endothelial function, could contribute to arterial elasticity.

The specific aims of this study were to evaluate the effect of pregnancy on carotid artery elasticity and to clarify if there were associations between maternal lipids, endothelial function and arterial elasticity during pregnancy.

## Methods

### Subjects

The Cardiovascular Risk in Young Finns is an ongoing population-based –center follow-up study of atherosclerosis risk factors in Finnish children and adolescents. The first cross-sectional survey was conducted in 1980. The original invited sample size was 4,320 children and adolescents aged 3, 6, 9, 12, 15 and 18 years. The individuals were randomly chosen from the national register. There were 3,596 participants. In 2001, 2,283 of these individuals were re-examined when they were aged 24–39 years. Out of this sample, 62 of the participants were pregnant and 62 non-pregnant women matched for age and smoking status were chosen as controls. In 2007 we re-examined 2204 (age 30-45) of these individuals and 37 women were pregnant. Again, 37 matched women were chosen as controls. In both 2001 and 2007, 10% of women in both pregnant and control groups were smoking and 90% were non-smokers. The smokers were defined according to the information participant self gave. Pregnant women who continued smoking and smoked at least one cigarette daily during pregnancy were classified as smokers. A total of nine women of the year 2007 sample were studied also in 2001, three of them being pregnant both times, three of them being pregnant once and three of them being controls in both times. Thus, there were 99 controls and 99 pregnant women in our study: 33 women (33.3%) in the first trimester (≤15 weeks), 18 in 2001 and 15 in 2001; 32 women (32.3%) in the second trimester (16–28 weeks), 25 in 2001 and 7 in 2007 and 29 women (29.3%) in the third trimester (≥29 weeks), 17 in 2001 and 12 in 2007. The gestational age data were not available for 5 subjects (5.1%): 2 patients in 2001 and 3 patients in 2007 and those subjects were excluded. After these exclusions 94 pregnant women were included, 29 (31%) of them being nulliparous. Participants provided written informed consent and the study was approved by the Ethics committee of the Hospital District of Southwest Finland.

Height and weight were measured. Blood pressure was measured with a random zero sphygmomanometer (Hawksley & Sons Ltd, Lancin, UK) while seated after 5 min rest and the average of three measurements was used in the analysis. For determination of serum lipoprotein levels venous blood samples were drawn after an overnight fast. All lipid determinations were conducted using standard methods as previously described [13].

### Carotid artery studies

Ultrasound studies were performed using Sequoia 512 ultrasound mainframes (Acuson, CA, USA) with 13.0 MHz linear array transducers. Left carotid artery was scanned following a standardized protocol. The intima-media thickness (IMT) was measured as previously described [14].

The assessment of carotid artery elasticity indexes was conducted by a method previously described [15]. The best quality cardiac cycle was selected from the 5-second clip images. The common carotid diameter 10 mm from carotid bifurcation was measured from the B-mode images using ultrasonic calipers at least twice in end-diastole and end-systole, respectively. The mean of the measurements was used as the end-diastolic and end-systolic diameter. Ultrasound and concomitant brachial blood pressure measurements were used to calculate the indexes of arterial elasticity. Blood pressure was measured just before and immediately after carotid artery ultrasound scanning. In the calculation mean of these values was used.

Young’s Elastic Modulus (YEM) gives an estimate of arterial stiffness that is independent of wall (intima-media) thickness [16] by the formula: ([systolic blood pressure – diastolic blood pressure] × diastolic diameter)/([systolic diameter – diastolic diameter]/IMT). Carotid artery distensibility (CAD) measures the ability of the arteries to expand in response to the pulse pressure caused by cardiac contraction and relaxation and was calculated as: ([systolic diameter – diastolic diameter]/diastolic diameter)/(systolic blood pressure – diastolic blood pressure). Stiffness Index (SI) is considered to be relatively independent of blood pressure [17] and was calculated by the formula: ln (systolic blood pressure/diastolic blood pressure)/([systolic diameter – diastolic diameter]/diastolic diameter).

In the assessment of brachial FMD, the left brachial artery diameter was measured both at rest and after reactive hyperemia. Increased flow was induced by inflation of a pneumatic tourniquet placed around the forearm to a pressure of 250 mmHg for 4.5 min, followed by release. Three measurements of arterial diameter were performed at end-diastole at a fixed distance from an anatomic marker, first at rest and then at 40, 60, and 80 s after cuff release. The vessel diameter in the scans after reactive hyperemia was expressed both as the change in absolute diameter (FMD) and as the percentage relative to the resting scan (FMD%) [18].

All statistical calculations were performed with the SPSS for Windows programs (SPSS, Chicago, IL). Statistical significance of difference between the groups was analysed by One-Way ANOVA. Univariate correlations were performed using the Pearson correlation. Stepwise multivariate analysis with linear regression was used to determine independent predictors of SI, YEM and CAD. Data are shown as mean ± SD. A P-value <0.05 was considered statistically significant.

## Results

Clinical characteristics, lipid profiles, blood pressure measurements and stiffness parameters of the non-pregnant and pregnant groups by trimester are shown in Table 1. In both groups, there were 10 smokers (10%). In the first trimester group the mean weight, body mass index (BMI), total cholesterol, low density lipoprotein (LDL) and triglycerides were lower than the corresponding values in the second or the third trimester groups and in the controls. According to group comparisons, all lipid values were in an increase throughout the pregnancy.

**Table 1:** Clinical characteristics, lipid profiles, blood pressure measurements and stiffness measures of the non-pregnant and pregnant groups by trimester

**Variable ± SD**	**Non-pregnant (N = 99)**	**First trimester (N = 33)**	**Second trimester (N = 32)**	**Third trimester (N = 29)**	**p-value**
Age (years)	32.2 ± 4.8	31.2 ± 4.0	31.6 ± 4.2	32.9 ± 5.7	0.330
Height (cm)	166.3 ± 6.6	166.4 ± 5.2	166.7 ± 5.0	165.5 ± 6.7	0.740
Weight (kg)	68.4 ± 13.5	65.4 ± 8.6	72.8 ± 11.8	77.3 ± 15.3	0.002
Gestation weeks		11.3 ± 3.3	23.3 ± 4.4	33.2 ± 2.9	
BMI (kg/m^2^)	24.7 ± 4.9	23.7 ± 3.6	26.2 ± 3.9	27.9 ± 4.9	0.002
Total cholesterol (mmol/l)	5.0 ± 0.8	4.6 ± 0.7	6.3 ± 1.0	6.9 ± 1.1	<0.001
LDL (mmol/l)	3.1 ± 0.8	2.6 ± 0.5	3.6 ± 0.8	3.9 ± 1.0	<0.001
HDL (mmol/l)	1.4 ± 0.3	1.5 ± 0.2	1.7 ± 0.3	1.9 ± 0.4	<0.001
Triglycerides (mmol/l)	1.1 ± 0.5	1.2 ± 0.5	2.1 ± 1.0	2.7 ± 1.0	<0.001
SBP (mmHg)	116 ± 13	111 ± 10	112 ± 13	113 ± 15	0.836
DBP (mmHg)	71 ± 10	64 ± 7	65 ± 9	71 ± 13	0.032
SI	5.50 ± 2.44	5.15 ± 1.67	6.47 ± 2.34	8.07 ± 3.13	<0.001
YEM (mmHg/mm)	900 ± 439	805 ± 278	1030 ± 428	1412 ± 695	<0.001
CAD (%/10 mmHg)	2.24 ± 0.87	2.43 ± 0.82	1.99 ± 0.84	1.56 ± 0.59	<0.001
CCD (mm)	5.42 ± 0.42	5.39 ± 0.40	5.60 ± 0.44	5.87 ± 0.50	<0.001
IMT (mm)	0.58 ± 0.08	0.57 ± 0.08	0.56 ± 0.08	0.55 ± 0.07	0.488
BBD (mm)	3.03 ± 0.32	3.05 ± 0.29	3.25 ± 0.25	3.27 ± 0.33	<0.001
FMD%	10.02 ± 4.41	9.56 ± 4.54	9.99 ± 4.90	10.23 ± 5.22	0.883

All values are mean ± SD. ANOVA- test between the three trimesters. *BMI*, body mass index; *LDL*, low density lipoprotein cholesterol; *HDL*, high density lipoprotein cholesterol; *SBP*, systolic blood pressure; *DBP*, diastolic blood; *SI*, stiffness index; *YEM*, Young’s elastic modulus; *CAD*, carotid artery distensibility; *CCD*, carotis communis diameter; *IMT*, carotid intima- media thickness; *FMD*, flow mediated dilation; *BBD*, baseline brachial artery diameter.

SI was 57% and YEM 75% higher and CAD 36% lower in the third trimester group than in the first trimester group. The diameter of the common carotid artery grew by 9% from the first to the third trimester whereas IMT remained equal in all groups. A decreasing tendency in carotid elasticity with advancing gestational weeks was seen also when the year 2001 and the year 2007 samples were examined separately (data not shown). The difference in FMD% between pregnancy groups was significant after adjustment for baseline brachial artery diameter: P = 0.033 for linearity between the three trimesters and P = 0.009 between all the groups. The main results were similar in nulliparas and multiparas (data not shown).

**Figure 1:**
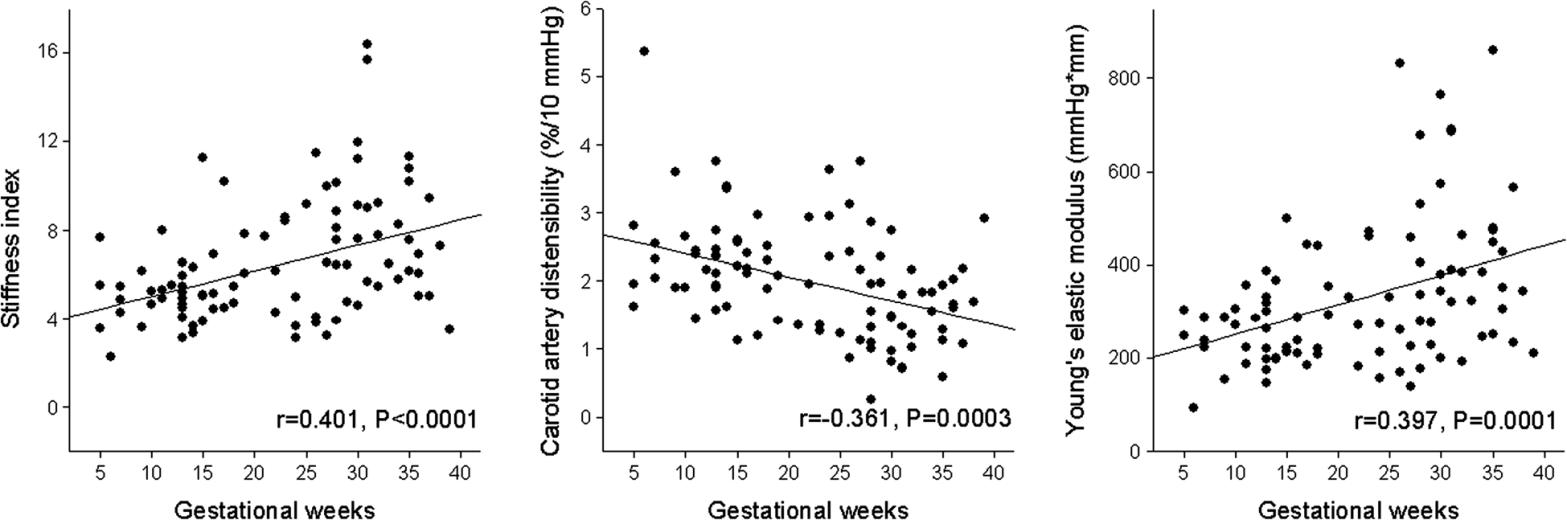
**Correlations between carotid elasticity indexes and gestational weeks.** The levels of the indexes in the control group are described in Table 1.

The correlations between variables reflecting clinical characteristics and carotid artery elasticity indexes are presented in Table 2. The strongest correlations of arterial elasticity were detected with gestational age (Figure 1), but also maternal age, weight, BMI, lipids and blood pressure seemed to correlate with stiffness indexes. Brachial artery FMD did not correlate significantly with any of the parameters reflecting carotid artery elasticity, but the baseline brachial artery diameter did. In non-pregnant women, SI, YEM and CAD correlated significantly with age, triglyceride levels and diameter of the common carotid artery. In the stepwise multivariate analysis, only gestational age correlated independently with all the arterial elasticity indexes (SI, YEM and CAD) (Table 3). YEM and CAD also strongly correlated with systolic blood pressure. In the control group, the stiffness indexes correlated with age, diameter of the common carotid artery, triglyceride concentrations and systolic blood pressure. Even after adjustment for the carotid artery diameter, the results of multivariate analyses remained unchanged.

**Table 2:** Pearson’s correlation coefficients

	**During pregnancy**					**Controls**		
	**Stiffness index**	**Carotid artery distensibility**	**Young’s elastic modulus**	**Flow mediated dilation**	**Stiffness index**	**Carotid artery distensibility**	**Young’s elastic modulus**	**Flow mediated dilation**
Gestational weeks	0.401**	-0.361**	0.427**	0.082				
Age (years)	0.294**	-0.309**	0.233**	-0.027	0.349**	-0.341**	0.322**	0.155
Weight (kg)	0.304**	-0.375**	0.326**	0.065	0.119	-0.167	0.127	0.203*
Height (cm)	0.058	-0.066	0.074	-0.221*	-0.137	0.100	-0.153	0.097
Systolic BP (mmHg)	0.223*	-0.443**	0.420**	0.108	-0.016	-0.241*	0.172	0.148
Diastolic BP (mmHg)	0.150	-0.264*	0.350**	0.112	0.022	-0.234*	0.203*	0.133
TC (mmol/l)	0.379**	-0.363**	0.330**	0.172	0.058	-0.011	0.023	0.033
LDL (mmol/l)	0.340**	-0.307**	0.281**	0.169	0.012	0.026	-0.004	0.109
HDL (mmol/l)	0.271**	-0.163	0.249*	0.102	-0.058	0.025	-0.092	-0.161
TG (mmol/l)	0.209*	-0.289**	0.203	0.085	0.277**	-0.179	0.253*	-0.042
BMI (kg/m^2^)	0.242*	-0.328**	0.236*	0.148	0.194	-0.214*	0.207*	0.162
CCD (mm)	0.246*	-0.305**	0.278**	-0.066	0.289**	-0.224*	0.265**	-0.126
IMT (mm)	-0.136	0.044	-0.347**	-0.059	-0.056	0.083	-0.296**	0.131
BBD (mm)	0.347**	-0.327**	0.316**	-0.275**	0.160	-0.121	0.170	0.263**

*BP*, blood pressure; *TC*, total cholesterol; *LDL*, low density lipoprotein cholesterol; *HDL*, high density lipoprotein cholesterol; *TG*, triglycerides; *BMI*, body mass index; *CCD*, carotis communis diameter; *IMT*, carotid intima- media thickness; *BBD*, baseline brachial artery diameter.

**Table 3:** Multivariate correlates of stiffness index, carotid artery distensibility and Young’s elastic modulus

	**Stiffness index**		**Carotid artery distensibility**		**Young’s elastic modulus**	
	**β**	**P value**	**β**	**P value**	**β**	**P value**
Pregnant women						
Gestational age	0.407	0.003**	-0.501	<0.001***	0.459	<0.001
Age	0.170	0.197	-0.192	0.082	0.057	0.567
Body mass index	0.064	0.651	0.009	0.942	0.014	0.899
Systolic BP	0.168	0.203	-0.675	<0.001***	0.318	0.002**
Diastolic BP	-0.064	0.643	0.362	0.024*	-0.206	0.109
TC	0.113	0.609	0.048	0.786	-0.039	0.814
LDL	0.031	0.886	0.054	0.739	-0.093	0.509
HDL	0.138	0.341	0.001	0.992	0.215	0.056
Triglycerides	-0.026	0.887	0.047	0.758	-0.223	0.118
CCD	0.124	0.359	-0.093	0.423	0.121	0.248
Controls						
Age	0.325	<0.001***	-0.317	0.001**	0.300	0.001**
Body mass index	0.053	0.573	-0.145	0.120	0.082	0.399
Systolic BP	-0.111	0.223	-0.195	0.037*	0.091	0.328
Diastolic BP	-0.136	0.144	0.069	0.693	0.075	0.436
TC	-0.074	0.433	0.054	0.565	-0.102	0.294
LDL	-0.074	0.417	0.090	0.969	-0.084	0.371
HDL	-0.002	0.983	0.013	0.892	-0.041	-0.447
Triglycerides	0.259	0.005**	-0.141	0.130	0.236	0.011*
CCD	0.283	0.002**	-0.203	0.029*	0.259	0.006**

Stepwise multiple-regression analysis; *P < 0.05;**P < 0.01; ***P < 0.001.

## Discussion

In the present study, we found that carotid artery elasticity decreased towards the end of the pregnancy and this was not correlating with the maternal hyperlipidemia or the diameter of the carotid artery. Furthermore, it was not mediated through pregnancy-related changes in endothelial function. All lipid levels tended to increase through pregnancy.

Previously it has been shown that aortic elasticity increases during pregnancy while carotid compliance decreases [8]. Accordingly, we observed a decrease in carotid artery elasticity during pregnancy. We also wished to evaluate possible mechanisms behind pregnancy-related changes in arterial elasticity, and therefore we analyzed the associations between arterial elasticity indexes and serum lipids and endothelial function. Although we detected a significant univariate correlation between arterial elasticity and maternal hyperlipidemia, in multivariate analysis after adjustment for gestational age, lipids were no longer significantly associated with arterial elasticity. Therefore, our study indicated the lack of an association between maternal hyperlipidemia and decreasing carotid artery elasticity. In both groups, but especially in the pregnant group, systolic blood pressure correlated with YEM and CAD, as also in previous studies [19].

In clinical terms, despite decreasing compliance in carotid arteries, the incidence of stroke is not elevated during pregnancy according to recent studies [20]. Pregnant women are young and usually quite healthy with apparently healthy carotid arteries and therefore a stroke during pregnancy is highly unlikely in spite of the decreased elasticity. However, the incidence of pregnancy-related stroke appears to be increasing, especially during the postpartum period and therefore the present findings have potential clinical relevance [21]. In our study there were no changes in the values of carotid intima-media thickness suggesting that the hyperlipidemia during pregnancy is not significantly atherogenic. This is an expected finding because even in the presence of major risk factors, e.g. in patients with the metabolic syndrome, the rate of thickening of intima-media complex is still so slow that atherosclerotic changes cannot develop to any measurable extent during a mere few months exposure [22]. In the study of Koskinen and co-workers, IMT progression was only 13 μm/year even in patients with the metabolic syndrome. During 9 months of pregnancy corresponding rate of IMT thickening could not result in marked changes in IMT.

However, some conflicting studies of carotid compliance during pregnancy have been published; Hu et al. reported that in women with uncomplicated pregnancies SI remained unchanged but the compliance increased [23]. In our study, the women in the first trimester of pregnancy had more compliant carotid arteries than the controls, the stiffening started to become apparent only in the second and third trimesters. Arterial elasticity increased in early pregnancy to accommodate increasing maternal cardiac output and blood volume, which occurred to some extent also in the carotid arteries in uncomplicated pregnancies as shown also by Spaanderman et al. [24]. In their study the last examination was carried out at 7 weeks of pregnancy with no follow-up data to determine what happened to carotid artery elasticity towards to the end of the pregnancy. In our former study [11] the endothelial function determined by FMD was slightly reduced during the first trimester and then increasing towards to the end of pregnancy, i.e. a mirror image of the findings in carotid artery distensibility. In that study the post-release diameter was measured at pre-defined time points, while with continuous measurements the result could have been different [25]. In line with our previous report we found a tendency towards improvement in the endothelial function with increasing weeks of pregnancy. Inspite of an improvement in the endothelial function we observed a significant decrease in the carotid artery elasticity. This means that endothelial function may not affect significantly with changes in carotid artery elasticity during pregnancy. Yuan et al. recently published a study with quite similar findings to ours, but they also studied some of the patients 20 months postpartum, showing recovery in stiffness indices and carotid diameter after delivery [26].

The reason of decreasing distensibility in carotid arteries and thus conflicting findings in comparison to observations related elasticity from other arteries during pregnancy is not known. However, it can be speculated that it is some kind of preserving effect where the pregnancy-related increases in cardiac output and blood volume with probable unfavorable effects on brain perfusion are kept in check through regulated carotid artery compliance. According to this hypothesis, a local decrease in carotid artery elasticity may actually be an appropriate adaptation to the altered hemodynamics occurring during pregnancy. Interestingly, Visontai et al. have speculated that the amount of estrogen receptors could be smaller in carotid arteries and this might explain the difference in elasticity from other arteries [7].

As carotid diameter affects carotid elasticity under normal conditions, we evaluated whether the pregnancy-related increase in stiffness would be due to increasing blood volume towards the end of pregnancy, i.e. whether the artery was dilated to its extremity. Therefore the analyses related to pregnant women were also adjusted for the common carotid artery diameter. However, this adjustment did not modulate any associations between elasticity indexes and possible correlates suggesting that the pregnancy-related decrease in carotid artery elasticity was not attributable to vessel wall distension caused by the increase in blood volume.

Our study population consisted of pregnant women from a large national cohort. Therefore we had no further information of potential preexisting disorders of the women or any possible complications in pregnancies such as gestational hypertension and gestational diabetes mellitus. On the other hand, the preexisting disorders could also have been present in the control group. We had no possibility to follow the subjects longitudinally, which of course was a limitation, and therefore we can only report associations between the exposure and outcome. We had also two recruitment times, 6 years apart and this can also be considered a weakness, although the analyses made separately in both 2001 and 2007 were similar. We did the analysis also excluding nine women who were studied twice but the results remained unchanged.

## Conclusions

Our observations reveal that the carotid artery stiffens during pregnancy but this is not correlated to the level of maternal hyperlipidemia or to changes in endothelial function in other parts of the maternal arterial tree.

## Abbreviations

CAD: Carotid artery distensibility; YEM: Young’s elastic modulus; SI: Stiffness index; FMD: Flow mediated dilation; T: Trimester; IMT: Carotid intima- media thickness; BMI: Body mass index; LDL: Low density lipoprotein cholesterol; HDL: High density lipoprotein cholesterol; SBP: Systolic blood pressure; DBP: Diastolic blood; CCD: Carotis communis diameter; BBD: Baseline brachial artery diameter; TC: Total cholesterol; TG: Triglycerides; BP: Blood pressure.

## Competing interests

The authors declare that they have no competing interests.

## Authors’ contributions

All authors participated in concepting and designing the study. HK performed the analysis and interpreted them with ToL, TiL and SH HK, ToL and SH participated in drafting the manuscript. All authors have revised the manuscript critically for important intellectual content and read and approved the final manuscript.

## Pre-publication history

The pre-publication history for this paper can be accessed here:

http://www.biomedcentral.com/1471-2393/14/98/prepub

## References

[B1] Khalil A Jauniaux E Cooper D Harrington K Pulse wave analysis in normal pregnancy: a prospective longitudinal study PLoS One 2009 4 7 e6134 10.1371/journal.pone.0006134 19578538 PMC2700961

[B2] Macedo ML Luminoso D Savvidou MD McEniery CM Nicolaides KH Maternal wave reflections and arterial stiffness in normal pregnancy as assessed by applanation tonometry Hypertension 2008 51 4 1047 1051 10.1161/HYPERTENSIONAHA.107.106062 18259025

[B3] Robb AO Mills NL Din JN Smith IB Paterson F Newby DE Denison FC Influence of the menstrual cycle, pregnancy, and preeclampsia on arterial stiffness Hypertension 2009 53 6 952 958 10.1161/HYPERTENSIONAHA.109.130898 19398652

[B4] Karkkainen H Heiskanen N Saarelainen H Valtonen P Lyyra-Laitinen T Laitinen T Vanninen E Heinonen S Ambulatory arterial stiffness index is unchanged in uncomplicated third-trimester singleton and twin pregnancies Acta Obstet Gynecol Scand 2011 90 5 516 523 10.1111/j.1600-0412.2011.01101.x 21501122

[B5] Ronnback M Lampinen K Groop PH Kaaja R Pulse wave reflection in currently and previously preeclamptic women Hypertens Pregnancy 2005 24 2 171 180 10.1081/PRG-200059871 16036401

[B6] Tihtonen KM Koobi T Uotila JT Arterial stiffness in preeclamptic and chronic hypertensive pregnancies Eur J Obstet Gynecol Reprod Biol 2006 128 1–2 180 186 16530917 10.1016/j.ejogrb.2005.12.026

[B7] Visontai Z Lenard Z Studinger P Rigo J Jr Kollai M Impaired baroreflex function during pregnancy is associated with stiffening of the carotid artery Ultrasound Obstet Gynecol 2002 20 4 364 369 10.1046/j.1469-0705.2002.00820.x 12383319

[B8] Mersich B Rigo J Jr Besenyei C Lenard Z Studinger P Kollai M Opposite changes in carotid versus aortic stiffness during healthy human pregnancy Clin Sci (Lond) 2005 109 1 103 107 10.1042/CS20040352 15740457

[B9] Brizzi P Tonolo G Esposito F Puddu L Dessole S Maioli M Milia S Lipoprotein metabolism during normal pregnancy Am J Obstet Gynecol 1999 181 2 430 434 10.1016/S0002-9378(99)70574-0 10454696

[B10] Mazurkiewicz JC Watts GF Warburton FG Slavin BM Lowy C Koukkou E Serum lipids, lipoproteins and apolipoproteins in pregnant non-diabetic patients J Clin Pathol 1994 47 8 728 731 10.1136/jcp.47.8.728 7962626 PMC502146

[B11] Saarelainen H Laitinen T Raitakari OT Juonala M Heiskanen N Lyyra-Laitinen T Viikari JS Vanninen E Heinonen S Pregnancy-related hyperlipidemia and endothelial function in healthy women Circ J 2006 70 6 768 772 10.1253/circj.70.768 16723801

[B12] Herrera E Ortega-Senovilla H Disturbances in lipid metabolism in diabetic pregnancy - Are these the cause of the problem? Best Pract Res Clin Endocrinol Metab 2010 24 4 515 525 10.1016/j.beem.2010.05.006 20832733

[B13] Juonala M Viikari JS Hutri-Kahonen N Pietikainen M Jokinen E Taittonen L Marniemi J Ronnemaa T Raitakari OT The 21-year follow-up of the Cardiovascular Risk in Young Finns study: risk factor levels, secular trends and east-west difference J Intern Med 2004 255 4 457 468 10.1111/j.1365-2796.2004.01308.x 15049880

[B14] Raitakari OT Juonala M Kahonen M Taittonen L Laitinen T Maki-Torkko N Jarvisalo MJ Uhari M Jokinen E Ronnemaa T Akerblom HK Viikari JS Cardiovascular risk factors in childhood and carotid artery intima-media thickness in adulthood: the Cardiovascular Risk in Young Finns Study JAMA 2003 290 17 2277 2283 10.1001/jama.290.17.2277 14600186

[B15] Juonala M Jarvisalo MJ Maki-Torkko N Kahonen M Viikari JS Raitakari OT Risk factors identified in childhood and decreased carotid artery elasticity in adulthood: the Cardiovascular risk in Young Finns study Circulation 2005 112 10 1486 1493 10.1161/CIRCULATIONAHA.104.502161 16129802

[B16] Riley WA Barnes RW Evans GW Burke GL Ultrasonic measurement of the elastic modulus of the common carotid artery: the atherosclerosis risk in communities (ARIC) Study Stroke 1992 23 7 952 956 10.1161/01.STR.23.7.952 1615543

[B17] Hirai T Sasayama S Kawasaki T Yagi S Stiffness of systemic arteries in patients with myocardial infarction: a noninvasive method to predict severity of coronary atherosclerosis Circulation 1989 80 1 78 86 10.1161/01.CIR.80.1.78 2610739

[B18] Juonala M Viikari JS Laitinen T Marniemi J Helenius H Ronnemaa T Raitakari OT Interrelations between brachial endothelial function and carotid intima-media thickness in young adults: the Cardiovascular Risk in Young Finns study Circulation 2004 110 18 2918 2923 10.1161/01.CIR.0000147540.88559.00 15505080

[B19] Urbina EM Srinivasan SR Kieltyka RL Tang R Bond MG Chen W Berenson GS Correlates of carotid artery stiffness in young adults: the Bogalusa Heart Study Atherosclerosis 2004 176 1 157 164 10.1016/j.atherosclerosis.2004.04.023 15306189

[B20] Scott CA Bewley S Rudd A Spark P Kurinczuk JJ Brocklehurst P Knight M Incidence, risk factors, management, and outcomes of stroke in pregnancy Obstet Gynecol 2012 120 2 Pt 1 318 324 22825091 10.1097/AOG.0b013e31825f287c

[B21] Kuklina EV Tong X Bansil P George MG Callaghan WM Trends in pregnancy hospitalizations that included a stroke in the United States from 1994 to 2007: reasons for concern? Stroke 2011 42 9 2564 2570 10.1161/STROKEAHA.110.610592 21799174

[B22] Koskinen J Kahonen M Viikari JS Taittonen L Laitinen T Ronnemaa T Lehtimaki T Hutri-Kahonen N Pietikainen M Jokinen E Helenius H Mattsson N Raitakari OT Juonala M Conventional cardiovascular risk factors and metabolic syndrome in predicting carotid intima-media thickness progression in young adults: the Cardiovascular Risk in Young Finns study Circulation 2009 120 3 229 236 10.1161/CIRCULATIONAHA.108.845065 19581494

[B23] Hu S Leonard A Seifalian A Hardiman P Vascular dysfunction during pregnancy in women with polycystic ovary syndrome Hum Reprod 2007 22 6 1532 1539 10.1093/humrep/dem028 17369295

[B24] Spaanderman ME Willekes C Hoeks AP Ekhart TH Peeters LL The effect of pregnancy on the compliance of large arteries and veins in healthy parous control subjects and women with a history of preeclampsia Am J Obstet Gynecol 2000 183 5 1278 1286 10.1067/mob.2000.106750 11084578

[B25] Weissgerber TL Davies GA Tschakovsky ME Brachial artery flow-mediated dilation is not affected by pregnancy or regular exercise participation Clin Sci (Lond) 2011 121 8 355 365 10.1042/CS20110008 21564020

[B26] Yuan LJ Xue D Duan YY Cao TS Zhou N Maternal carotid remodeling and increased carotid arterial stiffness in normal late-gestational pregnancy as assessed by radio-frequency ultrasound technique BMC Preg Childbirth 2013 13 1 122 10.1186/1471-2393-13-122 PMC3669620 23710816

